# Happy 5th Birthday Biology Open

**DOI:** 10.1242/bio.027508

**Published:** 2017-07-15

**Authors:** Jordan W. Raff, Rachel Hackett

**Affiliations:** 1 Sir William Dunn School of Pathology, University of Oxford, South Parks Road, Oxford OX1 3RE, UK; 2 The Company of Biologists, Bidder Building, Station Road, Cambridge CB24 9LF, UK

Jordan is the César Milstein Chair of Molecular Cancer Biology in the Sir William Dunn School of Pathology at the University of Oxford, and is Editor-in-Chief of Biology Open (BiO). Rachel Hackett is Managing Editor at BiO.

##  

**Fig. 1.**
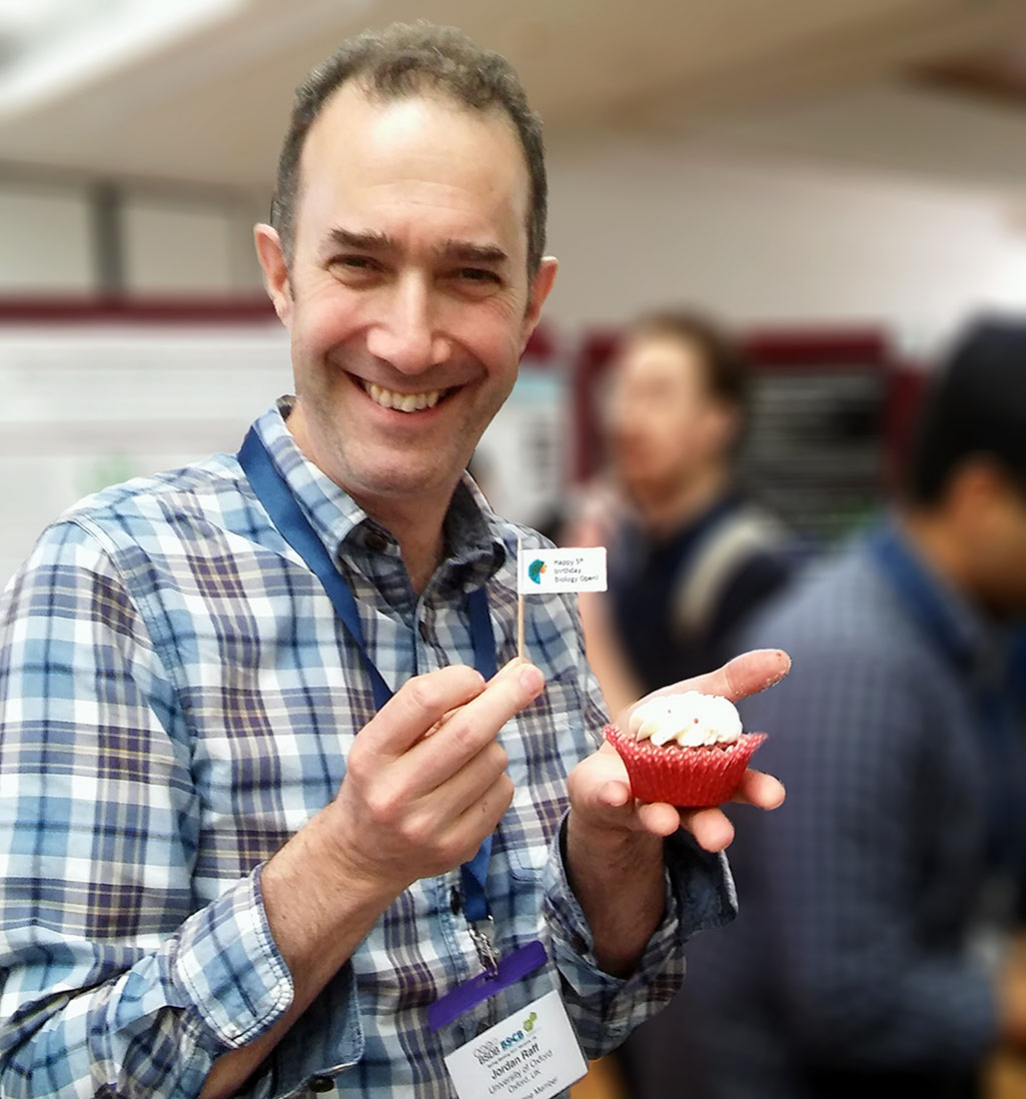
**Editor-in-Chief Jordan Raff celebrates BiO's birthday at the British Society for Cell Biology/British Society for Developmental Biology meeting in April 2017.**

The first issue of Biology Open (BiO) was published by The Company of Biologists, the long-established not-for-profit publishing organisation, in January 2012. The first article? An Editorial from our Editor-in-Chief outlining how BiO was going to reduce the pain to publish and make “life easier for us all” (Raff, 2012). In 2017, we celebrate five years of BiO (Fig. 1). So, six volumes, more than 60 issues and nearly 1000 published fully Open Access articles later, has BiO achieved its aims?

BiO was launched with an easy one-click online transfer system for authors whose papers were rejected by one of The Company of Biologists’ other journals – Development, Journal of Cell Science, Journal of Experimental Biology and Disease Models & Mechanisms. Transfer does not require reformatting and authors also have the opportunity to revise their article in response to any transferred reviews. In addition, BiO has a similar transfer agreement in place with the journal eLife.

It is, of course, also possible to submit articles directly to BiO; ∼50% of our authors do so. In April of this year, BiO joined the other Company of Biologists, journals in launching format-free submission to reduce the pain to submit for all our authors. We accept any format of paper (with references in any style) and ask you to do only what is absolutely necessary at submission. In practice, this means that other requirements will move to the revision stages – but we hope you won't mind at this point, given that we accept over 95% of revised manuscripts. As part of this change, and recognising the importance of reporting comprehensive materials and methods to aid transparency and reproducibility, we have removed the Materials and Methods section from our length limit to an article. BiO considers useful reports of negative results, but authors don't tend to submit them; perhaps busy researchers prioritise other papers?

It's not just authors who benefit from BiO. Articles transferred with existing reviews usually avoid the need for further rounds of review. This system speeds up editorial decisions and reduces the number of reviewers involved in the reviewing of such papers – reducing so-called ‘reviewer fatigue’ (Hunt and Moulton, 2012). For articles sent for review, BiO's streamlined review procedures are simple, fast, ethical and rigorous. Moreover, reviewers are asked not to suggest additional experiments unless these are absolutely necessary to support the main conclusions of the paper.

Speed has always been a focus for BiO, and is frequently reviewed and processes honed. Time to first decision is a key concern of authors. We have found speed of peer review the most difficult to improve, with busy reviewers struggling to meet the seven-day deadline. This leaves the Editor with a dilemma; wait for the existing reviewer or try someone else who may or may not be quicker or more appropriate. We're confident our Editors make the right decisions in most of these cases; BiO is quicker than its sister journals and key competitors, especially for articles transferred with reviews, but we are always striving for further improvement.

And what about speed after acceptance? To date, for 2017, the author's accepted version of their article appears online on average seven days after acceptance, with 38 days from acceptance to publication in an issue. And we will get faster too – in 2018, BiO moves to continuous publication, with the final versions of articles being published online as soon as they are ready, without having to wait for publication in a scheduled issue.

**Fig. 2.**
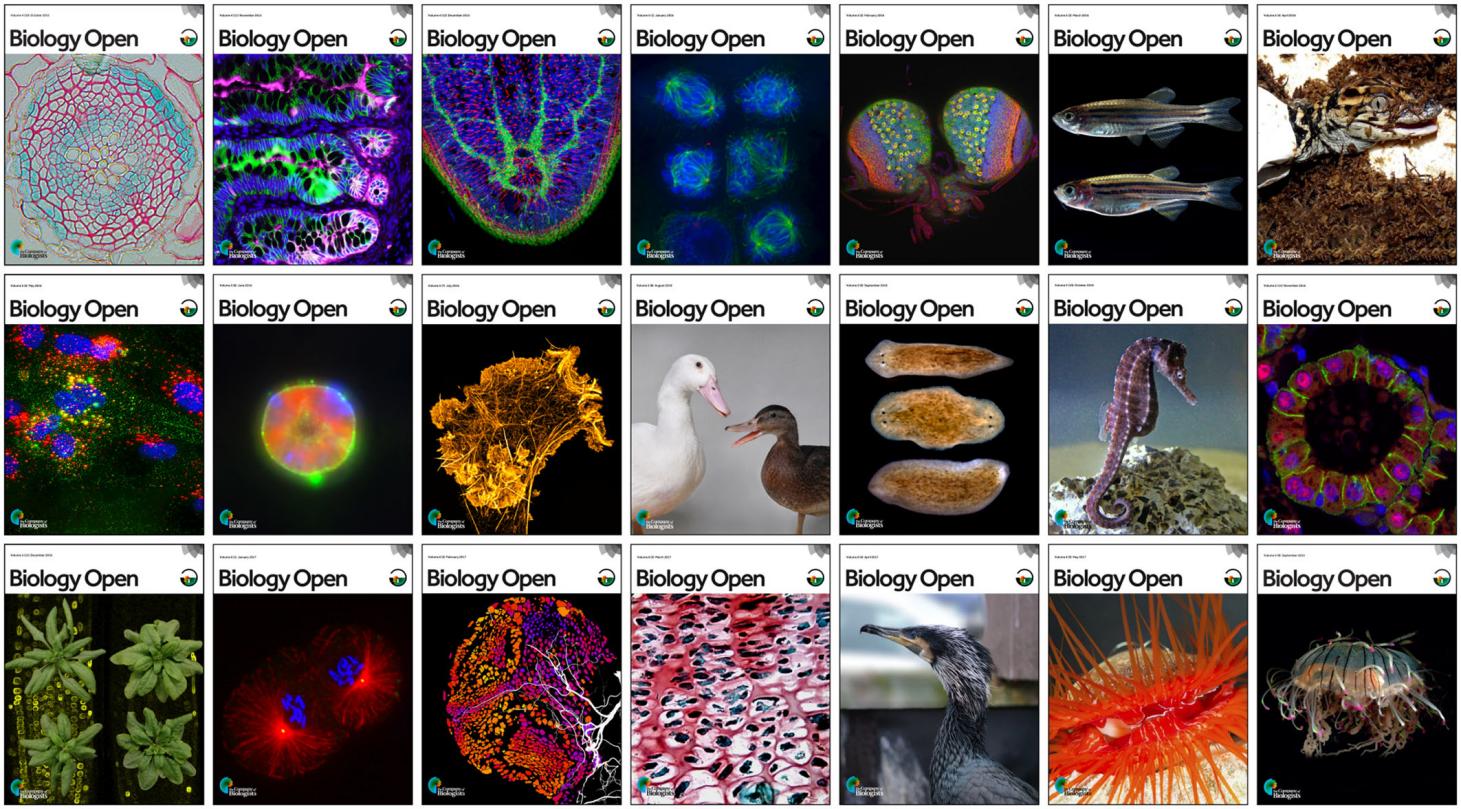
**BiO's rebranded covers showcase the diversity of its published research.**

Since its launch, BiO has strived to keep abreast of developments in the fast-moving field of STM (science, technology, medicine) publishing. As well as launching format-free submission, BiO has introduced free post-publication commenting in the form of eLetters. BiO recognises the value of widespread ORCID adoption and requires ORCID IDs to be provided at submission by corresponding authors. We launched a new enhanced web site in 2015 and underwent a rebranding at the same time (we like a challenge) (Fig. 2). BiO also supports preprints – authors submitting to BiO can simultaneously deposit their article in bioRxiv. And bioRxiv will return the favour – authors depositing a manuscript in bioRxiv can now transfer their paper directly to BiO. BiO publishes usage statistics and article-level metrics (Altmetrics) for all articles, giving authors the chance to see how publishing Open Access articles enhances the visibility of their research. BiO will continue to evolve and adopt new best publishing practices – always evaluating any benefit to authors, reviewers and readers.

The advent of Open Access publishing has proved to be good news for opportunists. The Open Access journals published by exploitative so-called ‘predatory publishers’ exist to make money. And presumably it works, otherwise why would we be seeing such growth in questionable journal practices worldwide? Reputable publishers and journals (such as BiO) can be identified by their inclusion in the Directory of Open Access Journals (DOAJ) and membership of OASPA (Open Access Scholarly Publishers Association). In addition, there are numerous ways in which it is possible to assess the validity of a journal, clearly outlined by the Think.Check.Submit initiative (http://thinkchecksubmit.org/).

Has BiO fulfilled its remit? For the authors of the nearly 1000 articles we have published, we certainly hope so. The feedback we receive from authors seems to indicate this, as does the number of repeat authors. Submissions to BiO have increased year on year, keeping our expert academic editors busy. BiO is committed to building on its sense of community with our authors, reviewers, editors and readers. As part of this, BiO and its sister journals fund a wide range of charitable activities that support the community of scientists in the areas covered by the journals. Why not publish with BiO and become part of our expanding community?
